# Mitochondrial Fission and Fusion in Tumor Progression to Metastasis

**DOI:** 10.3389/fcell.2022.849962

**Published:** 2022-03-09

**Authors:** Dillon P. Boulton, M. Cecilia Caino

**Affiliations:** ^1^ Department of Pharmacology, University of Colorado School of Medicine, Aurora, CO, United States; ^2^ Pharmacology Graduate Program, University of Colorado, Aurora, CO, United States

**Keywords:** mitochondria, cancer, metastasis, mitochondrial dynamics, fission, fusion

## Abstract

Mitochondria are highly dynamic organelles which can change their shape, via processes termed fission and fusion, in order to adapt to different environmental and developmental contexts. Due to the importance of these processes in maintaining a physiologically healthy pool of mitochondria, aberrant cycles of fission/fusion are often seen in pathological contexts. In this review we will discuss how dysregulated fission and fusion promote tumor progression. We focus on the molecular mechanisms involved in fission and fusion, discussing how altered mitochondrial fission and fusion change tumor cell growth, metabolism, motility, and invasion and, finally how changes to these tumor-cell intrinsic phenotypes directly and indirectly impact tumor progression to metastasis. Although this is an emerging field of investigation, the current consensus is that mitochondrial fission positively influences metastatic potential in a broad variety of tumor types. As mitochondria are now being investigated as vulnerable targets in a variety of cancer types, we underscore the importance of their dynamic nature in potentiating tumor progression.

## Introduction

Mitochondria are endosymbiont membrane bound organelles that carry out aerobic respiration to convert carbon fuels into cellular energy. The fact that mitochondria produce energy in the form of ATP is well known. However, mitochondria do more than “just” produce ATP; they participate in calcium (Ca^2+^) and iron homeostasis, redox balance, biosynthesis and programmed cell death. Moreover, mitochondria support a wide range of signaling pathways and regulate epigenetics.

The importance of mitochondria to cellular and organismal homeostasis is underscored by the fact that mitochondrial dysfunction has been associated with a wide range of human pathologies, including neurodegenerative disease, metabolic diseases, aging and cancer (Diaz-Vegas et al., 2020). The cancer field dogma that tumors universally reprogram their metabolism to aerobic glycolysis (the Warburg effect) led to the misconception that mitochondria were either dysfunctional or non-functional in cancer (Vander Heiden et al., 2009). Indeed, some tumors were found to contain mutations that inactivated mitochondrial respiration (Gottlieb and Tomlinson, 2005; Selak et al., 2005; Anastasiou et al., 2011; Zong et al., 2016). However, most tumors show enhanced glycolysis due to the presence of oncogenic mutations in K-RAS, C-MYC, PI3K; or due to inactivation of the tumor suppressors PTEN and P53 (Zong et al., 2016). While cancer cells may have increased rates of glycolysis, current evidence supports that even in this context mitochondria are still the main source of ATP production (Zong et al., 2016). In addition, mitochondria remain critical for integrating a variety of metabolic and signaling pathways in cancer cells (Zong et al., 2016).

Functional studies to probe the importance of mitochondrial activity in cancer led to the realization that mitochondria are required for several of the hallmarks of cancer. For instance, mitochondrial oxidative phosphorylation (OxPhos) was crucial for stemness of glioblastoma cells and tumor progression in mice (Janiszewska et al., 2012). Furthermore, blunting mitochondrial respiration by depletion of mtDNA or depletion of the mitochondrial transcription factor TFAM led to lower tumorigenesis (Weinberg et al., 2010; Tan et al., 2015). In contrast, mitochondrial biogenesis driven by PGC-1α increased malignant properties of pancreatic ductal adenocarcinoma and breast cancer cells (Viale et al., 2014; Nimmakayala et al., 2021). Glutamine mitochondrial metabolism supported K-RAS V12-dependent transformation, lung tumorigenesis, and metabolic reprogramming downstream of C-MYC (Gao et al., 2009; Weinberg et al., 2010). Furthermore, lipid β-oxidation and mitochondrial OxPhos were crucial for tumor repopulation of regressed pancreatic tumors *in vivo* (Viale et al., 2014); for *in vivo* age-related resistance to BRAF/MEK inhibition of melanoma (Alicea et al., 2020); and for anoikis resistance and tumor progression in ovarian cancer (Sawyer et al., 2020).

The importance of mitochondrial reactive oxidative species (ROS) and mitochondrial Ca^2+^ and iron homeostasis is underscored by its relation to cell cycle progression through M phase (Hao et al., 2021), melanoma growth *in vivo* and breast cancer metastasis (Ramchandani et al., 2021). To maximize mitochondrial function, tumors exploit protein homeostasis mechanisms driven by autophagic degradation and recycling, translational control of mammalian target of rapamycin (mTOR), chaperones and proteases (Cole et al., 2015). Inhibition of either chaperones of the heat-shock protein of 90 kDa (HSP90) family that reside in mitochondria, or mitochondrial matrix proteases Caseinolytic Mitochondrial Matrix Peptidases Proteolytic Subunit/Chaperone Subunit X (CLPP/CLPX) impaired tumor cell invasion *in vitro* and metastasis *in vivo* (Caino et al., 2013; Seo et al., 2016). In recent studies, high-content ATP breast cancer cells showed increased metastatic capacity when compared to low-content ATP cells; and targeting mitochondrial OxPhos on high-content ATP cells blocked metastatic dissemination (Fiorillo et al., 2018; Fiorillo et al., 2021). Overall, mitochondrial function supports several tumor cell-intrinsic phenotypes that contribute to tumor progression and metastasis.

**FIGURE 1:**
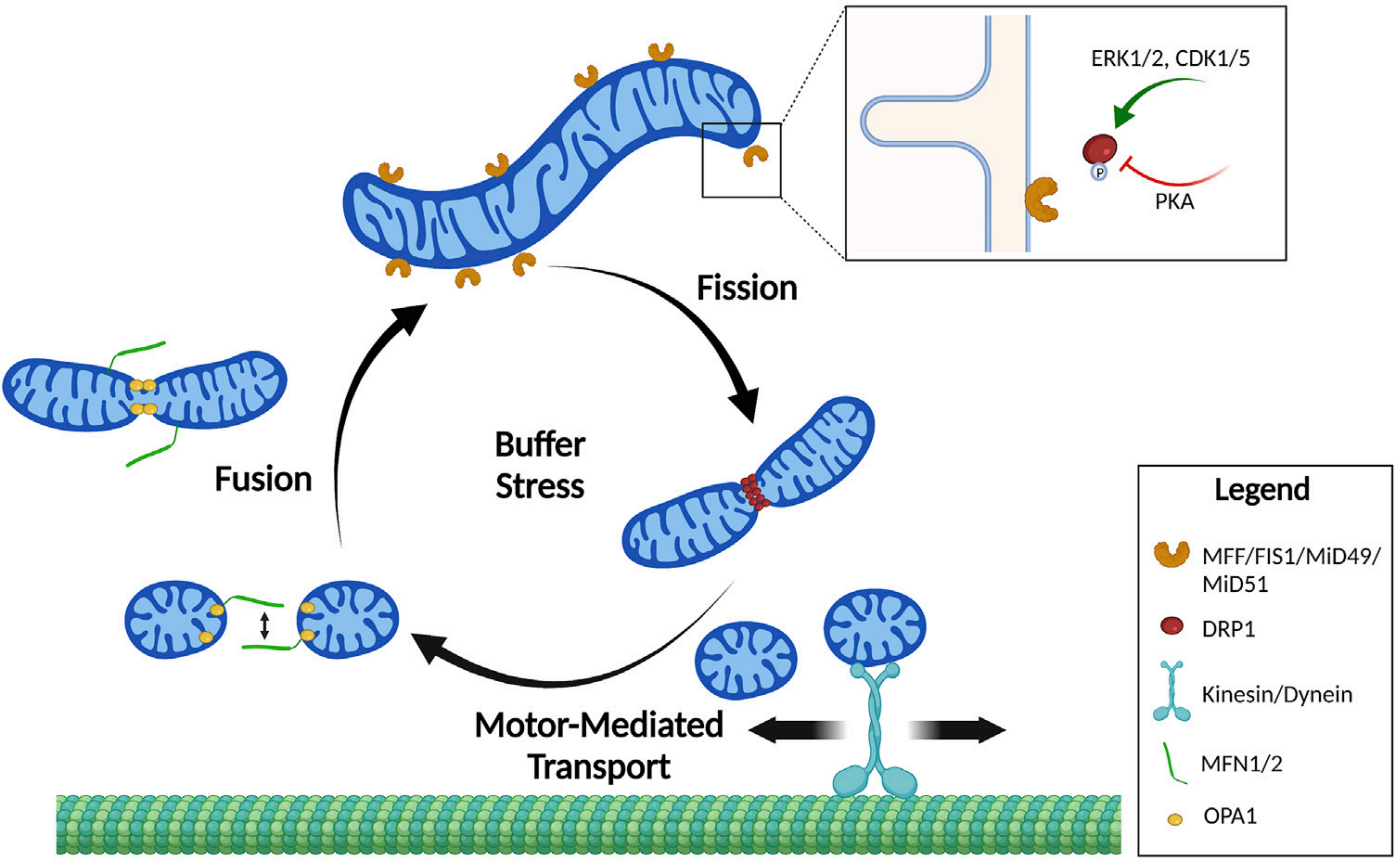
Mitochondrial shape cycles. Mitochondria constantly undergo dynamic changes in shape by the processes of fission and fusion. These shape changes are tightly regulated to buffer stress and localize mitochondria where they are needed. Mitochondrial fusion of the outer membrane is mediated by homo- and heterodimerization of MFNs. Fusion of the inner mitochondrial membrane is mediated by OPA1. Mitochondrial fission requires DRP1 recruitment to mitochondrial fission receptors: MFF, FIS1, MiD49, and MiD51. Mitochondrial fission is primarily regulated by phosphorylation of DRP1 by upstream kinases that increase (pS616) or decrease (pS637) DRP1 affinity to the receptors. Created in BioRender.com.

**FIGURE 2:**
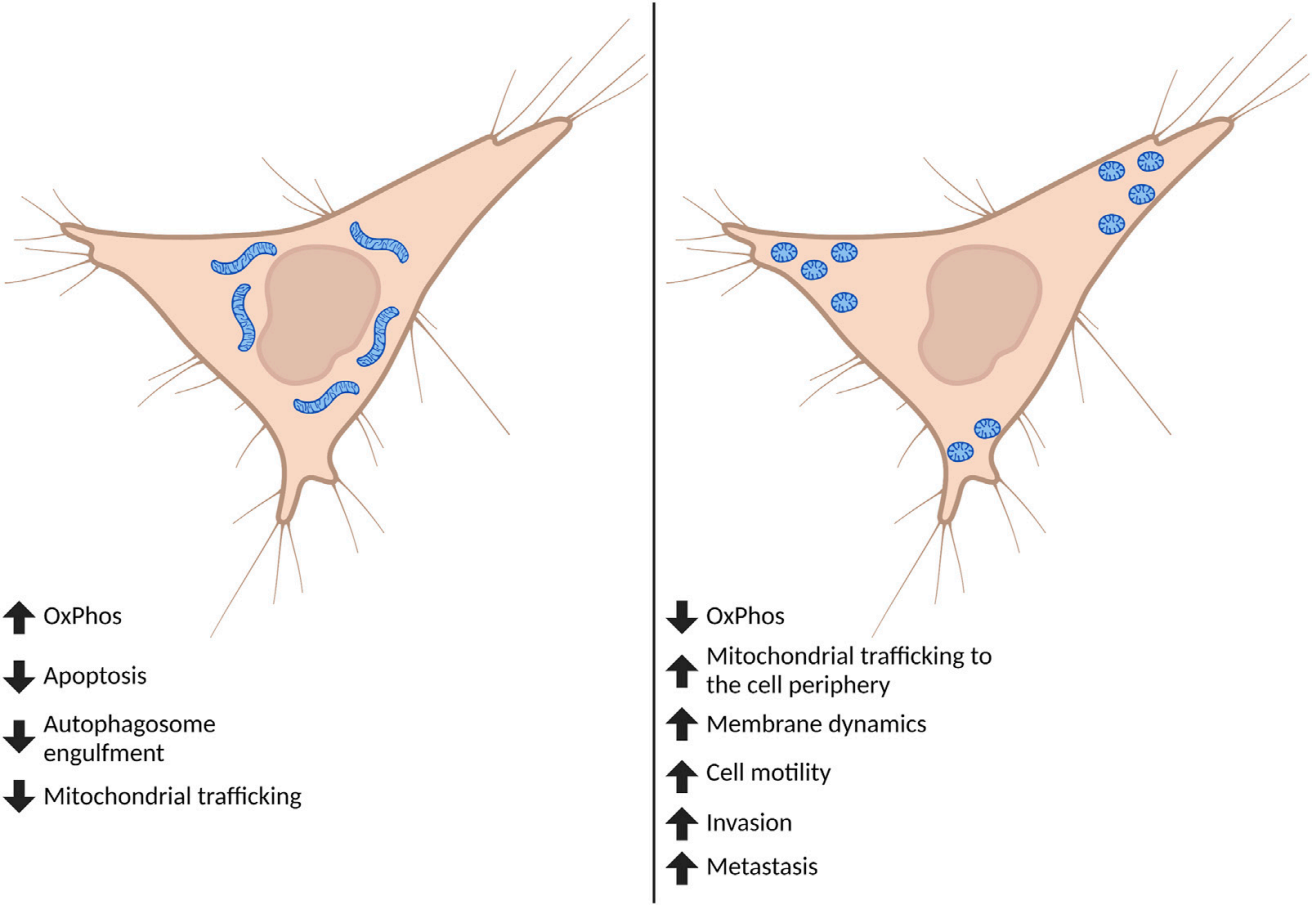
Effect of global mitochondrial shape on cell phenotypes. Cancer cells are often found to have fragmented mitochondria. Although mitochondrial fission and fusion undergo continuous cycles, when fission predominates this affects both mitochondrial and cell phenotypes important for the progression to metastasis. Highly fragmented pools of mitochondria can switch the metabolic phenotype of the cell and tend to accumulate at—and influence formation of—membrane protrusions including lamellipodia and invadopodia. The fragmentation of mitochondria ultimately drives increases in cell motility, invasion, and metastasis. Created in BioRender.com.

In order to adapt to different cellular, environmental, and developmental contexts, the cell maintains a heterogeneous pool of mitochondria in mechanisms collectively defined as mitochondrial dynamics (Osteryoung and Nunnari, 2003). Mitochondrial dynamics encompass many different processes that regulate gross mitochondrial morphology, ultrastructure of cristae, subcellular localization and crosstalk with other organelles (Youle and van der Bliek, 2012; Sheng, 2014). Gross mitochondrial morphology is controlled by mitochondrial fission—division of mitochondria—and mitochondrial fusion—the combination of two mitochondria into an individual elongated mitochondrion (Figure 1). Mitochondria undergo continuous cycles of fission and fusion in order to maintain a healthy pool of mitochondria and meet the metabolic demands of the cell. Imbalance in fission and fusion processes can lead to a fragmented or hyperfused pool of mitochondria, respectively. Hyperfused mitochondrial networks are often found to have enhanced OxPhos capacity, decreased mitochondrial trafficking, and are protected from lysosomal degradation during nutrient starvation (Osteryoung and Nunnari, 2003; Youle and van der Bliek, 2012; Sheng, 2014; Rambold et al., 2011; Gomes et al., 2011). Cells with fragmented mitochondria typically have enhanced glycolysis and are permissive for mitochondrial trafficking and mitochondrial degradation by macroautophagy (Osteryoung and Nunnari, 2003; Youle and van der Bliek, 2012; Sheng, 2014; Rambold et al., 2011; Gomes et al., 2011) (Figure 2). While mitochondrial size does not necessarily indicate pathology, recent evidence shows that mitochondrial fragmentation is associated with cancer and therapy resistance (reviewed in (Trotta and Chipuk, 2017)). However, the importance of fission and fusion in tumor progression to metastasis is far less known. Here, we will review the processes that regulate dynamic mitochondrial shape changes and emerging evidence that suggests their importance in tumor progression to metastasis.

### Section I: Mitochondrial Fission and Fusion in Cellular Homeostasis

Fission, or mitochondrial division, allows for the separation of daughter mitochondria (Wolf et al., 2019). When mitochondria are fully functional, fission serves as a process to increase the number of mitochondria within the cell and these mitochondria are permissive to be transported throughout the cell by molecular motors (Schwarz, 2013; Sheng, 2014). Interestingly, mitochondrial fission is cell cycle-regulated and during mitosis several kinases drive fragmentation of mitochondria to allow for their efficient distribution and inheritance to two daughter cells (Pangou and Sumara, 2021). Fission can also serve as an important component of mitochondrial quality control. When mitochondria become depolarized, fission in conjunction with intra-mitochondrial mechanisms of quality control and sorting provide a way to separate functional from dysfunctional components. The depolarized daughter mitochondria will activate signaling pathways to engage in mitochondrial selective autophagy, termed mitophagy (den Brave et al., 2021; Kleele et al., 2021). In this case, mitophagy will limit overproduction of ROS and potential release of pro-apoptotic factors from depolarized mitochondria (Poole and Macleod, 2021). While fission can serve as an important component of mitochondrial quality control, widespread fragmentation of the mitochondrial network is often associated with loss of mitochondrial membrane polarization and increased ROS production (Poole and Macleod, 2021).

Fusion is the combination of two or more individual mitochondria in which the outer and inner mitochondrial membranes fuse and the intermembrane space and matrix contents mix (Figure 1). Cells will utilize fusion as a key mechanism to mediate deleterious effects of damaged mitochondria to the cell. As an example, fusion of mitochondria with damaged ETC components or mtDNA mutations with healthy mitochondria will buffer ROS production, mitochondrial membrane depolarization and mitochondrial heteroplasmy (Youle and van der Bliek, 2012; Wallace and Chalkia, 2013; Giacomello et al., 2020). Thus, fusion is a key mechanism to adapt to cellular stress or mitochondrial damage (Gomes et al., 2011; Rambold et al., 2011). As another example for physiological relevance of fusion, mitochondria will elongate in response to stimuli that induce macroautophagy—like nutrient starvation—in order to prevent engulfment by autophagosomes (Gomes et al., 2011; Rambold et al., 2011).

One of the earliest functional consequences associated with mitochondrial shape changes was the link with the metabolic state of cells (Figure 2). Early studies suggested that cells with fragmented mitochondria displayed a glycolytic phenotype, while cells with elongated and interconnected mitochondrial networks were more reliant on OxPhos (Cowdry, 1924). We discuss in the sections below the link between mitochondrial shape and metabolism in more detail. However, it is important to note that gross mitochondrial morphology is not the only way to control OxPhos efficiency, which also depends on cristae arrangement, ETC regulation and supercomplex formation. Mitochondrial shape changes are also crucial for maintaining Ca^2+^ homeostasis, endoplasmic reticulum (ER)-mitochondria contacts and signaling, autophagy and apoptosis (Kraus et al., 2021).

In addition to these changes, mitochondria can undergo shape transitions such as the formation of circular mitochondrial donuts (Liu and Hajnóczky, 2011). Mitochondrial donut formation under hypoxia-reoxygenation involves swelling, detachment from the cytoskeleton and auto-fusion. Functionally, donuts were associated with increased membrane potential recovery under conditions of stress (Liu and Hajnóczky, 2011). Mitochondrial donuts also appear in response to mitochondrial ROS production (Ahmad et al., 2013). In this case, donuts were found to increase cytosolic Ca^2+^ intake under conditions of oxidative stress. Similarly, mitochondrial donuts found in quiescent glioblastoma cells had increased capacity to intake Ca^2+^ and improve survival of stem cells (Aulestia et al., 2018). Overall, formation of mitochondrial donuts in response to stress leads to increased Ca^2+^ homeostasis of the mitochondrial pool, or maintenance of mitochondrial membrane potential, ultimately preventing Ca^2+^ overload or depolarization-induced cell death. However, the role of mitochondrial donuts in tumor biology remains unexplored.

Due to the importance of mitochondrial shape changes in maintaining a physiologically healthy pool of mitochondria, aberrant cycles of fission/fusion are often seen in pathological contexts (Eisner et al., 2018). Recent evidence shows that mitochondrial shape, size and localization regulate several of the hallmarks of cancer. For instance, mitochondrial shape dynamics have been linked to metabolic adaptation (Schrepfer and Scorrano, 2016), cell cycle progression (Qian et al., 2012), necroptosis (Basit et al., 2017), autophagy (Huang et al., 2016), tumor growth, tumor cell motility (Ferreira-da-Silva et al., 2015), invasiveness and metastasis (Zhao et al., 2013).

The role of mitochondrial shape changes as regulators of cancer biology is reviewed in (Trotta and Chipuk, 2017; Williams and Caino, 2018). Emerging evidence suggests that the contribution of the mitochondrial fission/fusion proteins to tumor cell biology is tumor type dependent and may reflect the genetic makeup, hormonal/growth factor context, tumor microenvironment conditions and therapy responses of the tumor (Williams and Caino, 2018). As a result, strategies aimed at inhibiting fission or fusion have been proposed depending on the context (Yin et al., 2021), and pharmacological modulators of fission or fusion have been developed. In this context, we know little about how mitochondrial shape associates with progression to metastasis.

### Section II: Molecular Regulation of Mitochondrial Shape

Mitochondrial shape changes are orchestrated at the molecular level by large GTPases of the dynamin-related protein family (DRP) (Kraus et al., 2021). DRP’s act through conformational changes induced by GTP-binding, oligomerization and modification of the shape of biological membranes. The DRP group includes dynamins and dynamin-like proteins, guanylate-binding proteins, optic atrophy protein 1 (OPA1), mitofusins 1 and 2 (MFN1 and MFN2). DRPs are involved in fission, fusion or tubulation of intracellular membranes, thus controlling the morphology of different organelles.

Mitochondrial fission is carried out by a single GTPase, dynamin related protein 1 (DRP1, also known as DNM1L). The current model for fission includes recruitment of DRP1 from cytosol to the mitochondrial outer membrane, via one of its many receptors (reviewed in (Kraus et al., 2021)). Receptors include Mitochondrial Dynamics Protein of 49 and 51 kDa (MiD49/MiD51, also known as MIEF2/MIEF1), mitochondrial Fission 1 (FIS1) and Mitochondrial Fission Factor (MFF). These DRP1 receptors act by providing docking sites that stabilize the DRP1 dimers on the mitochondrial surface. Contacts to the actin cytoskeleton and ER-mediated constriction follow, eventually leading to elongation of the mitochondrial membranes and DNM-mediated scission of daughter mitochondria. Additional receptors and regulatory proteins for fission/fusion have been recently identified. Indeed, the Mitocarta 3.0 annotation includes ∼30 accessory mitochondrial dynamic genes (Rath et al., 2021). Recent studies have shown that lysosomes and the trans-Golgi network are involved in the division of mitochondria, highlighting the importance of inter-organelle crosstalk for efficient mitochondrial fission (Wong et al., 2018; Nagashima et al., 2020). Interestingly, it appears that different types of fission exist, with MFF-mediated DRP1 recruitment leading to partition into two equal daughter mitochondria and increases in mitochondrial numbers (Kleele et al., 2021). A second mechanism involves DRP1 recruitment via FIS1, usually leading to asymmetrical fission and production of a smaller mitochondrion that was coupled to mitophagy or mitochondrial derived vesicles (MDVs) degradation (Kleele et al., 2021).

Fusion is achieved by outer membrane GTPases MFN1 and MFN2, plus a single inner membrane GTPase OPA1 (Schrepfer and Scorrano, 2016). MFNs work as homo or heterodimers to bring outer mitochondrial membranes closer together by trans-binding of MFN proteins on opposite mitochondria (Giacomello et al., 2020). It is important to note that MFN2 also has additional functions outside of mitochondrial fusion, including maintaining ER-mitochondria contacts that are crucial for mitochondrial signaling and mitochondrial membrane dynamics (de Brito and Scorrano, 2008).

Expression of these GTPases and receptors are often dysregulated in cancer (see next section and ref (Williams and Caino, 2018)). Conditions of cellular stress, such as oxidative stress or metabolic stress led to transcriptional induction of MFNs. Furthermore, mitochondrial stress signaling pathways control protein stability of DRP1, leading to fusion (Kashatus et al., 2015). Cellular stress and induction of c-Jun N-terminal kinase (JNK) activity, on the contrary, stimulate mitochondrial fission by phosphorylation of MFN2 which is coupled to proteasomal degradation (Leboucher et al., 2012). Finally, post-translational modifications (PTM) control the activity, localization or abundance of the GTPases (Table 1).

**TABLE 1:** An overview of identified post-translational modifications found on commonly studied fission/fusion proteins and their effect on mitochondrial morphology and quality control.

Post translational modification	Effect on mitochondrial shape	Mechanism	Ref
S616-DRP1	Mitochondrial fragmentation	Phosphorylation by ERK2	Kashatus et al. (2015)
S616-DRP1	Mitochondrial fragmentation	Phosphorylation by CDK1	Taguchi et al. (2007)
S616-DRP1	Mitochondrial fragmentation	Phosphorylation by CDK5	Rong et al. (2020)
S616-DRP1	Mitochondrial elongation	Dephosphorylation by DUSP6	Ma et al. (2020)
S616-DRP1	Mitochondrial fragmentation	Phosphorylation by PINK1	Han et al. (2020)
S637-DRP1	Mitochondrial fragmentation	Dephosphorylation by PGAM5	Yu et al. (2020)
S637-DRP1	Mitochondrial elongation	Phosphorylation by PKA	Chang and Blackstone, (2007)
S637-DRP1	Mitochondrial fragmentation	Dephosphorylation by calcineurin	Cereghetti et al. (2008)
S155-MFF	Mitochondrial fragmentation	Phosphorylation by AMPK	Toyama et al. (2016)
S172-MFF	Mitochondrial fragmentation	Phosphorylation by AMPK	Toyama et al. (2016)
Y38-FIS1	Mitochondrial fragmentation	Phosphorylation by MET	Yu et al. (2021)
T562-MFN1	Mitochondrial fragmentation	Phosphorylation by ERK	Pyakurel et al. (2015)
S86-MFN1	Mitochondrial fragmentation	Phosphorylation by PKC	Ferreira et al. (2019)
Ub-MFN1	Mitochondrial fragmentation	Ubiquitination by PARKIN	Burchell et al. (2013)
Ub-MFN2	Loss of ER-mitochondria contacts	Ubiquitination by PARKIN	McLelland et al. (2018)
Ub-MFN2	Decreases mitophagy	Deubiquitination by USP30	Wang et al. (2015)
S442-MFN2	Mitochondrial fragmentation	Phosphorylation by PINK1	Chen and Dorn, (2013)
S27-MFN2	Mitochondrial fragmentation	Phosphorylation by JNK	Leboucher et al. (2012)
C684-MFN2	Mitochondrial elongation	Oxidation by GSSG	Shutt et al. (2012)

Amongst PTMs, phosphorylation of DRP1 is by far the best studied mechanism of regulation of mitochondrial shape. DRP1 is phosphorylated at multiple sites, of which S616 is a well-characterized site that exacerbates the GTPase activity of DRP1 and increases its affinity to receptors, thus promoting more efficient fission. S616-DRP1 is phosphorylated by extracellular signal-regulated kinase (ERK1/2 (Kashatus et al., 2015; Serasinghe et al., 2015)) and cyclin-dependent kinase 1 (CDK1 (Taguchi et al., 2007)), thus providing a link between mitochondrial division, proliferation and cell division (Figure 1). Furthermore, the non-conventional cyclin—CDK5—mediates phosphorylation of S616-DRP1 during neuronal differentiation (Rong et al., 2020). S616-DRP1 is also phosphorylated by PTEN-induced kinase 1 (PINK1) to promote fission prior to mitophagy (Han et al., 2020). Dual Specificity phosphatase 6 (DUSP6) keeps S616-DRP1 phosphorylation levels low under normal conditions, but oxidative stress induces degradation of DUSP6, hyperphosphorylation of DRP1 and increased mitochondrial fragmentation (Ma et al., 2020). Thus, pS616-DRP1 is under the control of several cellular signaling pathways and leads to changes in mitochondrial shape in response to extracellular and intracellular stimuli.

A second serine site on DRP1, S637, acts as an inhibitory PTM on GTPase activity and interaction of DRP1 with its receptors. S637 is phosphorylated by protein kinase A (PKA, Figure 1) and dephosphorylated by the calcium-dependent phosphatase calcineurin, or Bβ2, a mitochondria-localized protein phosphatase 2A (PP2A) regulatory subunit (Flippo et al., 2020). Thus, mitochondrial shape can be controlled by GPCR/cAMP signaling (leading to inhibition of fission), or Ca^2+^-dependent activation of calcineurin (leading to de-repression of fission). A recent report shows that protein kinase B (AKT) signaling leads to phosphorylation of S637-DRP1 to prevent mitochondrial fission in stem cells (Cha et al., 2021). Interestingly, PGAM5 removes phosphorylation marks on S637-DRP1, leading to de-repression of fission (Yu et al., 2020). Despite this evidence, the importance of S637 phosphorylation of DRP1 to inhibit DRP1 recruitment to mitochondria was recently disputed (Yu et al., 2019).

Fresh evidence indicates that DRP1 receptors are also regulated by phosphorylation. MFF is phosphorylated at S155 and S172 by AMP-activated protein kinase (AMPK) under metabolic stress, leading to enhanced recruitment of DRP1 and stimulation of fission (Toyama et al., 2016). FIS1 is phosphorylated at Y38 by MET receptor tyrosine kinase (RTK), which increases recruitment of DRP1 to mitochondria, affinity of DRP1 to FIS1, and mitochondrial fission (Yu et al., 2021). Although other phosphorylation sites on DRP1 and its receptors exist as documented in PhosphoSite Plus (Hornbeck et al., 2015), there is a large gap in thoroughly characterizing the functional relevance of these sites.

Interestingly, mitochondrial fusion is also regulated by PTMs. For example, ERK1/2 phosphorylates T562-MFN1, which inhibits MFN1 oligomerization and mitochondrial fusion (Pyakurel et al., 2015). Thus, ERK1/2 coordinates mitochondrial fragmentation by simultaneously stimulating fission and inhibiting fusion. S86-MFN1 is phosphorylated by protein kinase C ß (PKCß) which also leads to inhibition of fusion (Ferreira et al., 2019). On the other hand, Parkin Ub of MFN1 leads to proteasomal degradation and increased fission during mitophagy (Burchell et al., 2013).

S442-MFN2 is coupled to non-degradative ubiquitination (Ub) by E3 ubiquitin ligase, PARKIN, and fusion inhibition. Because PINK1 is exclusively stabilized under depolarization of the mitochondrial membranes, pS442-MFN2 connects mitochondrial stress to enhanced fission and mitophagy (Chen and Dorn, 2013). In this context, ubiquitin specific peptidase 30 (USP30) was shown to remove Ub marks and restore mitochondrial fission (Wang et al., 2015). In addition, MFN2 Ub by PARKIN has additional consequences in disassembling ER-mitochondria contacts, prior to mitophagy (McLelland et al., 2018). JNK phosphorylation at S27-MFN2 coupled to Ub and proteasomal degradation are involved in inhibition of mitochondrial fusion (Leboucher et al., 2012). On the other hand, oxidation of C684-MFN2 upon oxidative stress leads to MFN2 oligomerization and fusion stimulation (Shutt et al., 2012). AMPK phosphorylates S442-MFN2 (Hu et al., 2021), in this case leading to mitochondrial fission. Thus, AMPK stimulates fission by simultaneously phosphorylating MFF and MFN2.

Several proteases control the fusogenic activity of OPA1, including Overlapping With The M-AAA protease 1 Homolog (OMA1) and YME1 Like 1 ATPase (YMEL1). Long OPA1 (L-OPA1) is cleaved to produce a shorter form, S-OPA1. L-OPA1 and S-OPA1 work together to promote maximal fusion and OxPhos capacity (Del Dotto et al., 2017). Evidence suggests that heterotypic interactions between L-OPA1 and cardiolipin stimulate fusion. Homotypic interactions between L-OPA1 prevent its pro-fusion activity, but S-OPA1 binding to L-OPA1 restores fusion, therefore both forms of OPA1 are required for maximal fusion rates (Giacomello et al., 2020). In addition to proteolysis, OPA1 is subject to acetylation, nitrosylation and O-linked N-acetylglucosamine glycosylation (Giacomello et al., 2020). However, the consequences of these PTMs in the context of metastasis are unknown.

While there has been tremendous progress in understanding the regulation of the molecular machinery for mitochondrial fission and fusion, there is a significant gap in our understanding of how these mechanisms are (dys)regulated in cancer. In the next sections, we will summarize the current status of the field, in terms of common alterations of mitochondrial shape in cancer and the signaling pathways that regulate these mitochondrial shape changes, with an emphasis on tumor progression to metastasis.

### Section III: Mitochondrial Fission Promotes Metastatic Progression

As mitochondria are multifunctional organelles and are critical for various cellular functions, it is unsurprising that the dysregulation of mitochondrial shape changes play a critical role in the progression to metastasis (Figure 2). Fragmentation of mitochondria is widely thought to promote tumor progression (Trotta and Chipuk, 2017). Indeed, fission promoting proteins—including DRP1 (Zhao et al., 2013), MFF (Seo et al., 2019), FIS1 (Karimi et al., 2021; Soares et al., 2019), and MiD49 (Zhao et al., 2020)—are often found to have increased expression in cancer compared to normal adjacent tissues (Table 2). In an elegant study, one group observed how changes in DRP1 and MFN1 expression drives invasive and migratory phenotypes (Zhao et al., 2013). Using human breast cancer tissue microarrays, it was found that DRP1 expression was progressively upregulated throughout tumor progression with the lowest expression in normal tissues and the highest in lymph node metastases (Zhao et al., 2013). Interestingly, this trend was also found using frequently studied breast cancer cell models, where in comparison to non-metastatic breast cancer cells (MCF-7), metastatic breast cancer cells (MDA-MB-231 and MDA-MB-436) had more fragmented mitochondria correlating with higher protein expression of pS616-DRP1 and DRP1, and decreased MFN1 (Zhao et al., 2013). Inhibition of DRP1 through siRNA or pharmacological inhibition with Mdivi-1—an established inhibitor of DRP1—reduced migratory and invasive phenotypes found in these highly metastatic breast cancer cell lines (Zhao et al., 2013). Additionally, overexpression of MFN1 or MFN2 greatly reduced migratory and invasive capacity of these cells, supporting the idea that metastatic breast cancer cells drive migration and invasion through fragmentation of the mitochondria pool.

**TABLE 2:** An overview of global changes in expression of commonly studied mitochondrial fission and fusion proteins and the effect on tumor progression. N/D (not determined in the study).

Tumor type	Fission/Fusion protein expression	Impact on tumor progression	Model	Ref
Breast cancer	Increased DRP1 expression throughout tumor progression	Increased tumor cell migration and invasion	Patient samples; human cell lines	Zhao et al. (2013)
Hepatocellular carcinoma	Increased DRP1 expression in metastasis vs primary tumor	High DRP1 correlated with worse relapse-free survival	Patient samples	Sun et al. (2018)
Gastric cancer	Increased FIS1 expression in cancer vs normal tissue	Patients with metastases have more FIS1 than patients without metastases	Patient samples	Karimi et al. (2021)
Melanoma	FIS1 expression positively correlated with Clark level and was higher in stage IVb/c compared to stage III and stage IVa patients	No correlation with nodal or distal metastasis	Patient samples	Soares et al. (2019)
Non-small cell lung cancer	MFF	N/D	Patient samples	Seo et al. (2019)
Ovarian cancer	Increased MiD49 expression in cancer vs normal tissue	High MiD49 expression correlated with worse overall and progression-free survival	Patient samples; human cell lines	Zhao et al. (2020)
Pancreatic cancer	Decreased MiD49 expression in cancer vs normal tissue	Low MiD49 expression trends with worse overall survival (*p* = 0.064)	Patient samples; human cell lines	Bai et al. (2020)
Hepatocellular Carcinoma	Decreased MFN1 expression in metastasis vs primary tumor	Low MFN1 correlated with worse relapse-free survival	Patient samples	Sun et al. (2018)
Melanoma	MFN2 expression positively correlated with Clark level and was higher in stage IVb/c compared to stage III and stage IVa patients	High MFN2 expression is correlated with lymph node and distal metastasis	Patient samples	Soares et al. (2019)

In direct agreement with J. Zhao *et al.* (Zhao et al., 2013), it was found that mitochondrial fragmentation was a critical driver in hepatocellular carcinoma (Sun et al., 2018). Analysis of primary tumor tissue and extrahepatic metastases from patient samples revealed that DRP1 had increased mRNA and protein expression at metastatic sites in comparison to the primary tumor, whereas MFN1 mRNA and protein expression was decreased at metastatic sites in comparison to the primary tumor. High DRP1 expression, low MFN1, or high DRP1/MFN1 expression were all correlated with worse relapse-free survival (Table 2). Furthermore, DRP1 was necessary and sufficient for formation of intrahepatic and lung metastasis in orthotopic nude mouse hepatocellular carcinoma tumor models (Sun et al., 2018). Similarly, another group found increased *DRP1* and decreased *MFN1* mRNA expression in primary tumors from hepatocellular carcinoma patients in comparison to normal adjacent tissues (Zhang et al., 2020). Lower MFN1 expression was found in hepatocellular carcinoma patients with more advanced disease and correlated with a worse overall and disease-free survival. To validate these findings, MFN1 was overexpressed or genetically ablated in a cell line models of metastatic hepatocellular carcinoma. Overexpression of MFN1 increased mitochondrial size correlating with decreased cell growth, migration, invasion, tumor growth and lung metastasis *in vivo*. Strengthening this data, depletion of MFN1 decreased mitochondrial size correlating with increases in cell growth, migration, invasion, tumor growth and lung metastasis *in vivo* (Zhang et al., 2020). Overall, patients with high expression of DRP1 and/or low expression of MFN1—which will promote a more fragmented pool of mitochondria—are often correlated with more advanced disease and worse prognosis.

While the role of DRP1 in metastasis is more frequently studied, it is becoming apparent that the expression of DRP1 receptors are also dysregulated in cancer. Two recent studies used patient samples to determine potential changes of FIS1 during tumor progression (Karimi et al., 2021; Soares et al., 2019) (Table 2). In gastric cancer, *FIS1* mRNA expression was significantly higher in the primary tumor in comparison to matched normal adjacent tissue (Karimi et al., 2021). Furthermore, patients who developed metastasis tended to have higher *FIS1* expression compared to patients who did not (Karimi et al., 2021). In another comparative study, FIS1 and MFN2 protein expression were analyzed in cutaneous, oral, and sinonasal melanomas (Soares et al., 2019). In cutaneous melanoma both FIS1 and MFN2 protein expression correlated with a higher Clark level—a staging measure of how deep melanoma has invaded in the skin. FIS1 expression was significantly higher in oral melanoma patients diagnosed with stage IVb/c disease in comparison to stage III and stage IVa patients. MFN2 expression was higher in sinonasal melanoma patients diagnosed stage IVb/c disease in comparison to stage III and stage IVa patients. Interestingly, FIS1 expression was not correlated with disease progression in sinonasal melanoma and MFN2 expression was not correlated with disease progression in oral melanoma. FIS1 was found to be most highly expressed in oral melanomas compared to sinonasal melanoma, whereas MFN2 showed higher expression in sinonasal melanoma when compared to oral melanoma. This may indicate that even within melanoma, subtypes may differentially rely on fragmented vs elongated mitochondria for tumor progression. Further studies will need to be performed to determine mechanistically why certain melanoma subtypes may rely more on MFN2 as opposed to FIS1. One explanation of why certain subtypes of melanoma may prefer fusion to fission is the requirement of OxPhos over glycolysis for metabolism. As these two subtypes of melanoma originate in different microenvironments, it is possible that the availability of nutrients and oxygen required for OxPhos may be vastly different between these two subtypes of melanoma, thus requiring utilization alternate metabolic pathways. Additionally, while higher expression of FIS1 and MFN2 found in these large-scale studies may suggest higher rates of fission and fusion, respectively, these types of studies fail to observe differences in mitochondrial shape. Thus, it is possible that upregulation of these proteins could influence functions of these proteins outside of mitochondrial shape changes. As an example, MFN2 has shown to be important in mitochondrial trafficking, PINK1/PARKIN dependent mitophagy (Gegg et al., 2010), and ER-mitochondria membrane contacts (de Brito and Scorrano, 2008).

Although it is generally accepted that mitochondrial fission is seen as promoting tumor growth and metastasis, the role of fission promoting receptor MiD49 (also known as MIEF2) in metastasis remains controversial. In ovarian cancer MiD49 mRNA and protein expression was increased in tumorigenic tissue in comparison to peritumor samples, and higher MiD49 expression correlated with worse overall and progression-free survival (Zhao et al., 2020) (Table 2). Indeed, ablation of MiD49 with siRNA increased mitochondrial length while reducing ovarian cancer cell growth, migration, invasion, tumor growth in and lung metastasis *in vivo*. In direct contrast, in the context of pancreatic cancer, MiD49 mRNA and protein expression was reduced in tumorigenic tissue and established cancer cell lines in comparison to paired normal tissue and non-malignant pancreatic cells, respectively (Bai et al., 2020) (Table 2). Despite having reduced MiD49 protein expression in comparison to normal pancreatic cells, authors were still able to deplete MiD49 expression in pancreatic cancer cell lines. In pancreatic cancer depletion of MiD49 via siRNA still increased mitochondrial size, however it increased cell growth, migration, and invasion. Additionally, overexpression of MiD49 reduced tumor growth and lung metastasis *in vivo*. Taken together, it seems that MiD49 has an oncogenic role in ovarian cancer whereas in pancreatic cancer MiD49 acts as a tumor suppressor. One explanation of these differences could be differences in ROS signaling as a result of MiD49 ablation. In the context of tumor biology, it is known that ROS functions as a dual-edged sword, where a moderate increase in cellular ROS activates many signaling pathways to promote cell growth and survival and large increases in ROS will result in cell death (Moloney and Cotter, 2018). Both groups found that removing MiD49 reduces total cellular ROS. In pancreatic cancer cells, increases in tumor cell invasion and migration caused by removal of MiD49 were reduced through addition of ROS, H_2_O_2_. Contrarily, in ovarian cancer cells it is proposed that MiD49 promotes tumor progression through a ROS induced AKT/mTOR signaling pathway (Zhao et al., 2021). Thus, MiD49 dependent increases in ROS on tumor cell intrinsic phenotypes play a tumor type dependent role.

Overall, it is apparent that many fission-promoting proteins are often higher in tumorigenic and metastatic patient samples when compared to normal tissues (Table 2). Expression of these proteins often correlates with worse patient outcomes—and drive many cellular phenotypes important for tumor progression like migration, invasion, primary tumor growth and metastasis *in vivo*—underscoring that mitochondrial fission processes may serve as an important therapeutic target for metastatic disease. Interestingly, mitochondrial shape changes do not require global changes in protein expression from all fission/fusion proteins and can be driven by select expression changes of one or two key proteins. Indeed, many studies show changes in only one or two fission/fusion proteins while observing no changes in others. As an example of this, in prostate cancer addition of exogenous androgens promoted the expression of DRP1 but not MFF, FIS1, MFN1, MFN2, or OPA1 (Lee et al., 2020). The proto-oncogene transcription factor, N-MYC, has been found to bind to promoter regions of DRP1 and control expression of this protein in Burkitt lymphoma and neuroblastoma cells, while not affecting other fusion proteins like MFN1 and OPA1 (Agarwal et al., 2019). Other groups have shown regulation of DRP1 and MiD49 expression via different miRNA, which directly control invasive and migratory capacity of cancer cells (Liang et al., 2020; Zhao et al., 2020). Thus, differential expression of select fission/fusion proteins could be explained by tumor type specific upstream regulatory factors, tumor microenvironment conditions, and hormone status (Williams and Caino, 2018). Furthermore, it is possible that many of these proteins are regulated via PTMs. For comprehensive studies—in addition to observing changes in total protein expression—it is critical to test for PTMs to DRP1 and its receptors as well as DRP1 recruitment to mitochondria.

### Section IV: Mitochondrial Fission Influences Metastasis Through Multiple Independent Mechanisms

While it is clear in many tumor types that proteins promoting fission often have increased expression, there is not simply one mechanism in which the fragmentation of mitochondria drive tumor progression. Indeed, mitochondrial shape changes influence metabolic changes (Liang et al., 2020; Zhang et al., 2020; Zhao et al., 2021), Ca^2+^ driven motility (Sun et al., 2018; Herkenne et al., 2020), and lamellipodia formation (Zhao et al., 2013) to promote tumor cell motility, invasion, and metastasis (Figure 2).

As key organelles in regulating cellular metabolism, mitochondria host a variety of metabolic processes including the citric acid cycle (TCA), oxidative phosphorylation, and ß-oxidation of fatty acids. It is known that mitochondrial shape changes can directly affect metabolism within the cell, reviewed in depth by Mishra and Chan (Mishra and Chan, 2016). Indeed, multiple independent groups have found that expression changes to fission/fusion proteins changes the metabolic programs of cancer cells which directly impacts their metastatic propensity. For example, in pancreatic cancer cells overexpression of DRP1—fragmenting the mitochondrial pool—resulted in an increase in glucose uptake and increased lactate production, suggesting a switch from oxidative (OxPhos) to glycolytic metabolism (Liang et al., 2020). While this did not directly test a change cellular metabolism, increases in migration and invasion caused by overexpression of DRP1 were reduced growing DRP1 overexpressing cells in galactose (which forces cells to rely on ATP generated via OxPhos). This suggests that in pancreatic cancer cells, increases in migratory and invasive capacities caused DRP1 dependent mitochondrial fragmentation requires glycolysis. Supporting the idea that fragmented mitochondria drive a change to a more glycolytic form of metabolism, it has been observed in hepatocellular carcinoma cells that fragmentation of mitochondria—via ablation of MFN1—reduces the oxygen consumption rate:extracelluar acidification rate (OCR/ECAR ratio) (Zhang et al., 2020). OCR/ECAR ratio describes relative utilization of glycolysis (ECAR) or OxPhos (OCR) for energy, where a higher OCR/ECAR ratio suggests cells rely more on OxPhos than glycolysis for ATP production. Furthermore, ablation of MFN1 with shRNA increased expression of many glycolytic enzymes, all together suggesting these cells switch to a more glycolytic form of metabolism (Zhang et al., 2020). This change in metabolism was important for mediating migratory and invasive capacity of cells as blocking glycolysis—via addition of 2-Deoxy-d-Glucose—reduced the increase in migration and invasion caused by ablation of MFN1.

Lastly, two recent publications describe the role of MiD49 in reprogramming ovarian cancer cell metabolism. Initial work by this group shows overexpression MiD49 reduced OCR and decreased ATP production concomitant with higher glucose uptake and lactate production. Mass-spectrophotometry analysis revealed MiD49 overexpressing cells had increased glycolytic intermediates and decreased TCA cycle intermediates in comparison to control cells (Zhao et al., 2020). Similar to results seen in DRP1 overexpressing pancreatic cancer cells, forcing MiD49 overexpressing cells to undergo OxPhos by supplementing media with galactose blocked increases in migration and invasion. In a follow up study, it was found that loss of MiD49 increased rates of cholesterol biosynthesis and *de novo* lipid synthesis but did not affect lipid uptake or fatty acid oxidation (Zhao et al., 2021). In elegant studies these authors show increased ROS as a result of MiD49 expression, activates mTOR/AKT signaling which drives the transcriptional activity of sterol regulatory binding protein 1 and 2 (SREBP1 and 2), to increase expression of critical genes involved in fatty acid and cholesterol synthesis. Indeed, increases in growth, migration, and invasion caused by overexpressing MiD49 could be attenuated by ablation of SREBP1 or SREBP2. Taken together, mitochondrial shape changes can dramatically alter mitochondrial dependent metabolism, which in turn promote many tumor cell intrinsic phenotypes critical for metastasis including growth, migration, and invasion.

Efficient cell migration requires a series of coordinated signaling events at the front of the cell, also known as the leading edge. Lamellipodia, invadopodia, and filopodia are different types of cellular protrusions observed at the leading edge of migrating cells and are critical for efficient cell movement, reviewed by Anne J. Ridley (Ridley, 2011). Mitochondrial shape changes have been shown to influence formation of these membrane protrusions in many tumor types, which mechanistically links fission/fusion cycles to migratory and invasive capacity of cancer cells. In hepatocellular carcinoma cells, fragmented mitochondria led to increased intrahepatic and lung metastasis, which could be dramatically reduced after treatment with Mdivi-1 (Sun et al., 2018). Mechanistically, fragmentation of mitochondria activated a Ca^2+^/CaMKII/ERK/FAK pathway that led to a decrease in focal adhesions and increased number of membrane protrusions. Elongating mitochondria with Mdivi-1 increased the number of focal adhesions and decreased membrane protrusions correlating with a dramatic reduction in tumor cell migration. This suggests that Ca^2+^ release as a result of mitochondrial fission activates a signaling cascade resulting in faster focal adhesion turnover and increase in membrane protrusions allowing for more efficient cell migration and metastasis. In agreement, another group observed elongation of mitochondria—*via* loss of DRP1 or overexpression of MFN1—dramatically reduced lamellipodia formation (Zhao et al., 2013). In a novel signaling pathway where receptor tyrosine kinase MET promoted mitochondrial fission through fission receptor FIS1, mitochondrial fission was critical for formation of lamellipodia and invadopodia formation (Yu et al., 2021). Indeed, knockout of either MET or FIS1 inhibited the cells’ ability to form lamellipodia or invadopodia cell protrusions, suggesting that mitochondrial fission is required for efficient formation of these cell protrusions.

Not only does mitochondrial fragmentation seem to induce cell protrusions, but it has become apparent that mitochondrial subcellular localization is extremely important for spatiotemporally fueling processes at the cell periphery that promote metastasis, reviewed in depth by Furnish and Caino (Cunniff et al., 2016; Schuler et al., 2017; Furnish and Caino, 2020). In addition to increases in lamellipodia and invadopodia formation seen by J. Zhao *et al.* (Zhao et al., 2013) and *Yan Yu et al.* (Yu et al., 2021), these groups also saw that more fragmented pools of mitochondria tended to have more mitochondria accumulated at sites of membrane protrusions. Recently, the role of epidermal growth factor receptor (EGFR) translocation to mitochondria was explored in the context of metastatic dissemination of non-small cell lung cancer (Che et al., 2015). Having found endogenous EGFR translocates to mitochondria upon stimulation with its ligand epidermal growth factor (EGF), authors used exogenous expression of synthetic construct of EGFR targeted to mitochondria, mitEGFR, to characterize the role mitochondrial EGFR. Expression of mitEGFR greatly increased lung metastasis of non-small cell lung cancer cells injected intravenously. Additionally, mitEGFR fragmented the pool of mitochondria, which accumulated in lamellipodia. While this only shows a correlation between mitochondrial size and accumulation in lamellipodia, it contributes to many other studies that have observed more fragmented mitochondria localized at cell protrusions.

Several recent papers provide evidence that defy canonical thinking that proteins promoting fusion will exclusively inhibit localization of mitochondria and subsequent invasive capacity of tumor cells. Using prostate cancer as a model, we showed that under conditions of extended PI3K inhibition mitochondria will adapt by trafficking to the cell periphery to mediate migration and invasion (Caino et al., 2015). In agreement with the concept that fragmented mitochondria will accumulate at cell protrusions, loss of MFN1 in cells grown in basal conditions greatly increased the number of mitochondria seen in cell protrusions. Interestingly, increases in mitochondria found in cell protrusions after PI3K inhibition required expression of MFN1. This suggests that mitochondrial trafficking to the periphery may require different fission/fusion components depending on the stimulus. It is also possible, that mitochondrial fusion at cortical regions of the cell would inhibit retrograde trafficking of mitochondria towards the nucleus. In another study, we identified the mitochondrially localized protein, syntaphilin (SNPH), as an important molecular brake for mitochondrial trafficking and metastasis (Caino et al., 2016). Depletion of SNPH resulted in mitochondria trafficking to the cell periphery and increased focal adhesion turnover resulting in dramatically increased cell motility and cell invasion. Furthermore, overexpression of SNPH in prostate cancer cells significantly reduced liver metastases. Similarly, expression of SNPH in melanoma cells reduced metastatic dissemination in an orthotopic syngeneic mouse model (Caino et al., 2017). Incredibly, we found that depletion of SNPH increased both mitochondrial fission and fusion events. Indeed, loss of MFN1 or MFN2 reduced the invasive capacity of cells lacking SNPH (Caino et al., 2016). Taken together, it appears that both mitochondrial fission and fusion are critical drivers of both formation of membrane protrusions as well as mitochondrial subcellular positioning, which drives cell motility, invasion, and metastasis.

While most groups claim that mitochondrial fission seems to be more important in forming membrane protrusions and mitochondrial repositioning, several recent papers provide conflicting data. One possible explanation is that most groups have observed changes in lamellipodia/filopodia formation under basal conditions, while conflicting evidence has been found in the context of protein overexpression systems and after treatment with potent kinase inhibitors. Thus, it is possible that the requirement of mitochondrial fission/fusion to mediate cell motility, invasion, and metastasis might be context dependent. Another explanation might be the method of quantification of mitochondrial fission/fusion. While many groups image and qualitatively “score” mitochondria based on gross morphology, usage of live cell video microscopy coupled to computational analysis may reveal changes in both fission and fusion rates. Indeed, there are multiple contexts where rates of mitochondrial fission and fusion rates were both increased (Caino et al., 2016; Wang et al., 2019a) correlating with increased cell motility and invasion. More work will need to be done to determine if the cells ability to quickly undergo either mitochondrial fission and/or fusion is more important for promoting metastasis than an imbalance in one direction in this cycle.

Most papers claiming the importance of mitochondrial shape changes in metastasis, focused on the tumor-cell intrinsic effect of this process. However, a recent landmark paper was published describing the importance of OPA1 in endothelial cells and how regulation of this protein in endothelial cells directly impacts tumor growth and metastasis (Herkenne et al., 2020). These authors found that after stimulation with vascular endothelial growth factor (VEGF), OPA1 protein expression was greatly increased and corresponded with a significant elongation of mitochondria. Deletion of OPA1 from the endothelial cells in mice fragmented the mitochondrial pool and reduced endothelial cell migration, proliferation, and overall growth. Reduction in the angiogenic capacity of the endothelial cells—mediated by an endothelial cell specific OPA1 knockout—significantly reduced primary tumor growth and metastasis in melanoma models. Interestingly, these authors found that simply fragmenting mitochondria in endothelial cells was not sufficient to reduce angiogenesis or primary tumor growth. Indeed, silencing of MFN1 or MFN2 fragmented mitochondria similar to silencing of OPA1, however there was no effect on the endothelial cell motility, proliferation, or growth. Additionally, knockout of MFN1 in endothelial cells did not affect melanoma primary tumor growth *in vivo*. Mechanistically, it was found that loss of OPA1 increased cytosolic Ca^2+^, activating NFκB, reducing key angiogenic gene expression, and inhibiting tumor vasculature leading to a significant reduction in tumor growth and metastasis. This study emphasizes two key points for studying mitochondrial shape changes in progression to metastasis: 1) fission and fusion proteins may have critical alternate functions outside of mediating mitochondrial size and 2) examining the importance of mitochondrial fission and fusion in alternate cell types. Indeed, in the context of endothelial cells, it appears that mitochondrial fragmentation and Ca^2+^ release actually inhibited the migratory ability of these cells. This is in direct contrast to Xiachen Sun *et al.* (Sun et al., 2018) who found in hepatocellular carcinoma cells that loss of MFN1 leading to mitochondrial fragmentation actually increased migratory and invasive capacity via a Ca^2+^/CAMKII/ERK/FAK pathway. Thus, mitochondrial fission has distinct consequences depending on the cell type that mitochondria are fragmented in and the protein that is altered to fragment mitochondria. While there have been many studies describing the importance of mitochondrial fission/fusion in regulating angiogenesis (Kim et al., 2018; Gӧbel et al., 2020), Stephanie Herkenne *et al.* (Herkenne et al., 2020) is the first paper to describe the functional effect of mitochondrial fission in endothelial cells in relation to how this impacts progression to metastasis. This work underscores the importance of studying how mitochondrial fission/fusion cycles in stromal or tumor microenvironmental cells impacts metastasis. More work will need to be done in the future to fully elucidate mechanisms in which mitochondrial fission/fusion in non-tumorigenic cells impacts tumor metastasis.

### Section V: Growth Factor and Kinase Signaling Drives Mitochondrial Fission to Promote Metastasis

**FIGURE 3:**
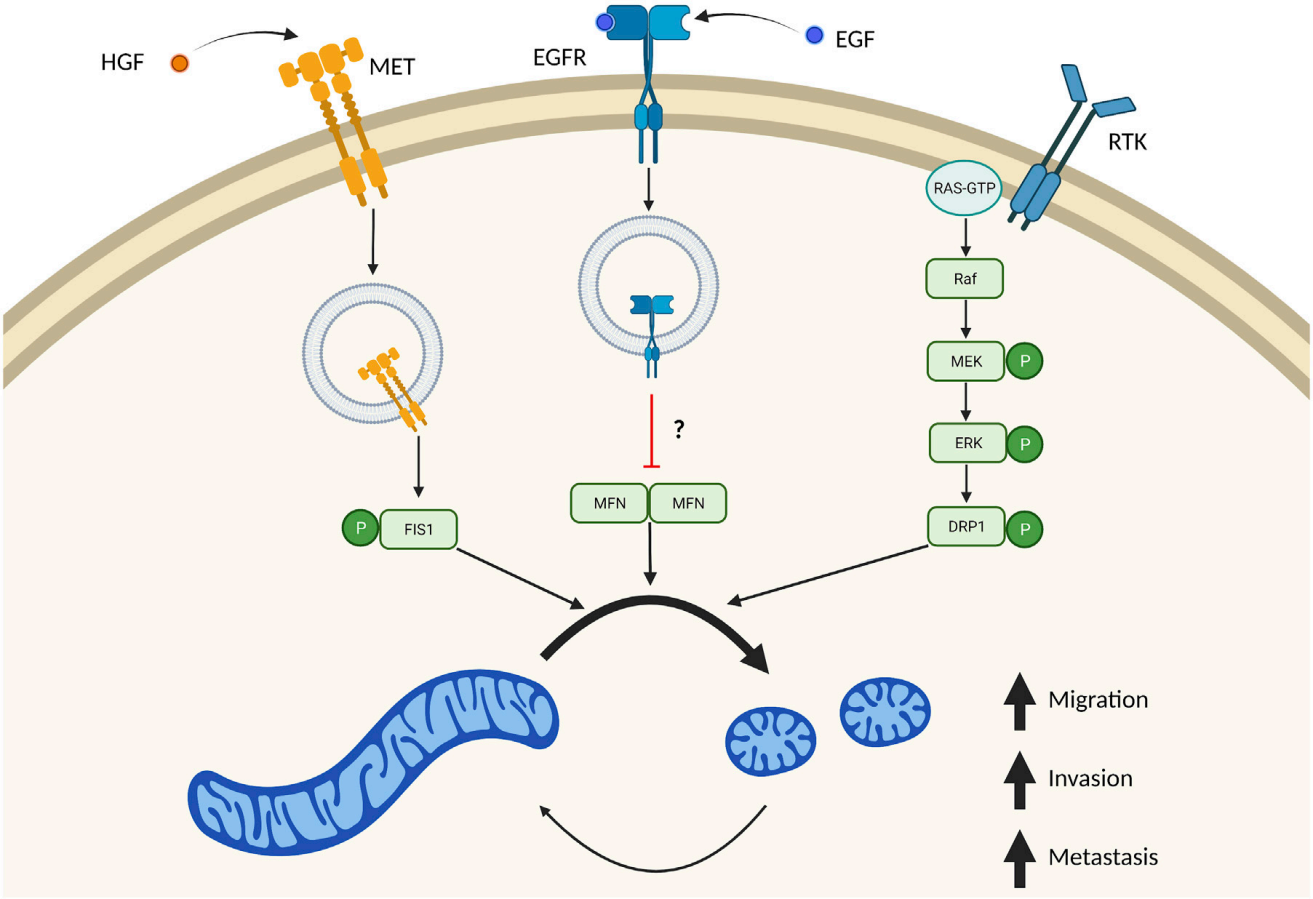
Multiple independent signaling pathways drive mitochondrial fission in metastasis. Many signaling pathways important in tumor biology also result in increased mitochondrial fission. These independent signaling pathways result in imbalanced fission/fusion cycles resulting in a highly fragmented pool of mitochondria. Stimulation of MET by HGF drives internalization of this receptor, where it directly phosphorylates FIS1. Phosphorylation of FIS1 by MET increases its affinity to DRP1, which drives mitochondrial fragmentation and tumor cell migration, invasion, and metastasis. EGF binding to EGFR also results in receptor translocation to mitochondria which blocks mitochondrial fusion. It remains unclear if this mechanism is through inhibition of MFN dimers. Overactive RAS signaling also drives mitochondrial fission through ERK1/2 dependent phosphorylation of DRP1. Created in BioRender.com.

Why are smaller mitochondria often observed in highly invasive and metastatic cancer cells? One main mechanism appears to be the effect of ERK1/2 on DRP1. It is known ERK1/2 1) promotes tumor progression, 2) is often hyperactivated due to frequently mutated RAS signaling and 3) promotes mitochondrial fragmentation through phosphorylating residue S616 on DRP1 (Kashatus et al., 2015; Serasinghe et al., 2015; Guo et al., 2020). In simultaneous publications, two groups identified direct phosphorylation of S616-DRP1 by ERK1 and ERK2 (Kashatus et al., 2015; Serasinghe et al., 2015). As previously mentioned, phosphorylation of S616-DRP1 promotes DRP1 recruitment to mitochondria and fragmentation of the mitochondrial pool. Here, they proposed that mitochondrial fission was driven via RAS mediated activation of ERK1/2, phosphorylation of DRP1, and subsequent fragmentation of mitochondria (Figure 3). These original studies looked at DRP1 phosphorylation by ERK1/2 in the context of RAS dependent growth and transformation. Since these publications there has been a growing body of evidence supporting a role of ERK1/2 dependent mitochondrial fission in promoting tumor growth and metastasis.

One study looked at SIRT4/ERK/DRP1 in non-small cell lung cancer tumor progression (Fu et al., 2017). SIRT4 is a mitochondrial localized deacetylase whose role is mainly described in controlling energy metabolism (Min et al., 2018). These authors found that SIRT4 had far lower protein expression in tumorigenic tissue compared to normal adjacent tissue. Furthermore, SIRT4 expression was found to be even lower in lymph node metastases in comparison to respective primary tumor. Overall, this suggests loss of SIRT4 positively influences tumor progression. Indeed, loss of SIRT4 increased non-small cell lung cancer cell growth, migration, and invasion. Mechanistically, depletion of SIRT4 promoted mitochondrial fragmentation through increased ERK1/2 dependent phosphorylation of DRP1. An increase in pDRP1 was found in lymph node metastases in comparison to primary tumor samples. Solidifying the role of this signaling pathway, authors found that inhibition of ERK—through inhibiting direct upstream kinase MEK—or DRP1 reduced increases in invasive capacity caused by loss of SIRT4. While this signaling pathway was not validated *in vivo*, this contribution strongly supports a role for activation of ERK driving mitochondrial fission leading to tumor progression.

Another study showed the role of a long non-coding RNA (lncRNA)—L22NC03-N14H11.1—in the tumor progression of hepatocellular carcinoma (Yi et al., 2020). L22NC03-N14H11.1 had increased expression in patients diagnosed with stage III/IV cancer compared to those diagnosed with stage I/II. In addition, patients who developed metastases had higher expression of L22NC03-N14H11.1 compared to those who didn’t. This suggests a strong role for this lncRNA in promoting tumor progression. Indeed, depletion of L22NC03-N14H11.1 reduced cell growth, migration, and invasion in hepatocellular carcinoma cells. Changes in these tumor cell-intrinsic phenotypes were driven by a decrease in pS616-DRP1 and could be rescued via overexpression of DRP1. Through extensive studies, it was found that L22NC03-N14H11.1 increased H-RAS signaling—and subsequent activation of ERK—by repressing transcription of a negative regulator of H-RAS expression, LZTR1. Taken together, this demonstrates the importance of lncRNA—L22NC03-N14H11.1—in promoting tumor progression by repressing a negative regulator of H-RAS. The increase in H-RAS expression via this lncRNA, is proposed to promote ERK dependent phosphorylation of DRP1.

Two recent studies look at how RAC GTPase activation protein 1 (RACGAP1) influences metastasis through ERK dependent mitochondrial fission and quality control (Ren et al., 2021; Zhou et al., 2021). RACGAP1 contains a conserved Rho GAP domain which facilitates the inactivation of small GTPases of the Rho family including RAC1, CDC42, and RHOA (Touré et al., 1998). In breast cancer, *RACGAP1* mRNA is expressed higher in tumorigenic tissue in comparison to normal tissue, and higher expression of *RACGAP1* leads to a worse overall survival (Ren et al., 2021). Intravenous tail injection of RACGAP1 overexpressing cells in MCF7 increased lung metastasis in comparison to control cells. Similarly, intravenous tail injection of MDA-MB-231 stably expressing RACGAP1 targeting shRNA decreased lung metastasis, demonstrating RACGAP1 expression positively correlates with lung metastasis *in vivo*. Invasive capacity of RACGAP1 overexpressing cells could be reduced via pharmacological elongation of the mitochondrial pool. Mechanistically it was found that pERK1/2 and pDRP1 increased with overexpression RACGAP1, both of which could be reduced via MEK inhibition (Ren et al., 2021). Strengthening this, MEK inhibition increased mitochondrial and subsequently decreased invasive capacity in a dose dependent manner. This suggests the importance of ERK1/2 in mediating mitochondrial fission to promote tumor cell invasion. In a follow up study, authors used breast cancer models to investigate the role of lncRNA, RACGAP1P, which acts as an endogenous competitor for a microRNA that degrades RACGAP1 (Wang et al., 2019b; Zhou et al., 2021). RACGAP1P was expressed higher in tumorigenic tissues in comparison to matched normal tissues. Patients with lymph node or distal metastasis tended to have higher expression of RACGAP1P, and this higher expression correlated with a worse overall survival (Zhou et al., 2021). Using two different cell models, authors found ectopic expression of RACGAP1P increased migration, invasion, and lung metastasis. Taken together, this provides a strong role for RACGAP1P for promoting tumor progression. RACGAP1P expression positively correlated with RACGAP1 expression, and as seen previously with RACGAP1 overexpression, overexpressing RACGAP1P resulted in increased pDRP1 and mitochondrial fragmentation. Elongation of mitochondria via Mdivi-1 or mitochondrial fusion promoter 1 (M1) reduced migration and invasion in RACGAP1P overexpressing cells, suggesting mitochondrial fragmentation is important for mediating these phenotypes. While authors suggest that mitochondrial fragmentation with increased RACGAP1P expression may be mediated through ERK, this was not tested. Overall, while these initial studies demonstrate an important role for RACGAP1 in and its associated lncRNA, RACGAP1P, in metastasis more work will need to be done to fully solidify the role of ERK dependent mitochondrial fragmentation in mediating metastatic phenotypes.

While many studies suggest ERK activation drives mitochondrial fission, a recent study in hepatocellular carcinoma cells suggests that, inversely, mitochondrial fission can also activate ERK1/2 (Sun et al., 2018). Here, it was proposed that overexpression of DRP1 increased cytosolic Ca^2+^, which activated a downstream signaling pathway involving CAMKII and ERK. Indeed, increases in pCAMKII and pERK in DRP1 overexpressing cells could be attenuated by treating cells with Ca^2+^ chelator, BAPTA-AM. While increases in cytosolic Ca^2+^ with DRP1 expression was never directly tested, rigorous studies demonstrate increases in cell migration associated with increased DRP1 expression was dependent on Ca^2+^, CAMKII, and ERK. Taken together, this shows the importance of an ERK/DRP1 signaling axis in promoting metastatic phenotypes. Interestingly, it appears this signaling axis may operate as a loop where ERK can promote mitochondrial fission through phosphorylation of DRP1, and mitochondrial fission may in turn have an impact on ERK activation.

Outside of ERK1/2 phosphorylation of DRP1, there are also several papers demonstrating receptor signaling as being critical in driving mitochondrial fission and promoting metastasis. EGFR is a receptor tyrosine kinase (RTK), that can be internalized upon binding to its ligand, including EGF (Goh and Sorkin, 2013). Internalization of these receptors can mediate new forms of signaling pathways (Lin et al., 2001). EGFR is a receptor tyrosine kinase (RTK), that can be internalized upon binding to its ligand, including EGF (Goh and Sorkin, 2013). Internalization of these receptors can mediate new forms of signaling pathways (Lin et al., 2001). A mitochondrial pool of EGFR has recently been described in regulating mitochondrial fission and promoting metastasis in non-small cell lung cancer cells (Che et al., 2015). Ectopic expression of a mitochondrial targeted EGFR (mitEGFR) dramatically increased migration, invasion, and *in vivo* lung metastasis of non-small cell lung cancer cells injected intravenously. Additionally, expression of mitEGFR or treatment with EGF resulted in fragmented mitochondria, via inhibition of mitochondrial fusion. Mechanistically, mitEGFR binds to MFN1 where it partially blocks MFN1 complexes. While it was not determined that the disrupted complexes were MFN1 dimers, it is proposed that mitEGFR facilitates cell migration by binding to MFN1, blocking dimerization with other MFN1 proteins, and directly inhibiting mitochondrial fusion (Figure 3). Indeed, mitEGFR dependent increases in cell migration could be reduced by overexpressing MFN1. Taken together, this proposes a novel signaling pathway where EGFR blocks mitochondrial fusion to drive metastatic propensity.

Recently an elegant study was published, where RTK MET was described in a novel signaling pathway with FIS1/DRP1 that promotes mitochondrial fragmentation and metastasis in hepatocellular carcinoma cells (Yu et al., 2021). Similar to stimulation of EGFR with EGF, stimulation of MET with ligand hepatocyte growth factor (HGF) localized MET to mitochondria. MET localization to mitochondria fragmented this mitochondrial pool consistent with more DRP1 at mitochondria. Using in depth biochemical analysis, authors identified a novel phosphorylation site (Y38) on FIS1 mediated by MET (Figure 3). pY38-FIS1 dramatically increases binding with DRP1 and rates of mitochondrial fission. In FIS1^−/−^ cells, re-expression of FIS1 dramatically increased lung metastasis and was fully dependent on phosphorylation of the Y38-FIS1. Additionally, it was proposed that this change in metastatic propensity is due to mitochondrial fission as treatment with Crizotinib—a MET inhibitor—or Mdivi-1 resulted in reduction of migratory and invasive potential to equal extents. While it is known that phosphorylation of mitochondrial fission receptors increases their affinity to DRP1, this is the first study to demonstrate the importance of these phosphorylation events in an *in vivo* metastatic model.

While most studies describing the role of DRP1 in tumor progression show that mitochondrial fission occurs through increases in total DRP1 or pS616-DRP1 expression, a recent study outlines the importance of the PKA-dependent phosphorylation of DRP1 at the serine 637 residue in inhibiting tumor cell motility (Aggarwal et al., 2019). As previously mentioned, phosphorylation of DRP1 at S637 inhibits DRP1 function and negatively regulates mitochondrial fission (Chang and Blackstone, 2007; Cereghetti et al., 2008). dAKAP1—a mitochondrial localized protein that binds PKA and protein phosphatase 1—controls bidirectional phosphorylation events by scaffolding the kinase and phosphatase to mitochondria. Indeed, dAKAP1 appears to be a negative regulator of metastasis as in breast cancer, metastatic samples had less dAKAP1 expression than paired primary tumor samples (Aggarwal et al., 2019). Interestingly, invasiveness of highly invasive breast cancer cell models negatively correlated with dAKAP1 mRNA and protein expression. Silencing of dAKAP1 dramatically reduced pS637-DRP1 after serum starvation—a known environmental stress that induces pS637-DRP1. Ablation of dAKAP1 also fragmented mitochondria and reduced mitochondrial fusion. Expression of dAKAP1 dramatically reduced migratory and invasive capacity of breast cancer cells. Interestingly, this required the PKA binding domain supporting the model that dAKAP1 scaffolds PKA to mediate phosphorylation of S637-DRP1, mitochondrial elongation, and a reduction in mitochondrial migration and invasion. Taken together this emphasizes the importance of negative regulators of DRP1 in mediating tumor progression. A recent paper demonstrated that phosphorylation of S637-DRP1 does not affect DRP1 localization to mitochondria and can rescue hyperfusion as a result of DRP1 knockout (Yu et al., 2019). While this goes against original studies, which describe pS637-DRP1 as a negative regulator of fission, it was found that phosphomimetic S637D-DRP1 was less efficient than phospho-null S637A-DRP1 or WT DRP1 at fragmenting mitochondria. Thus, it is still possible that dAKAP1 localization of PKA to mitochondria to mediate mitochondrial fragmentation through phosphorylation of S637-DRP1 is still a valid model.

Overall, it is clear that mitochondrial fission is important for driving many factors important for tumor progression including cell motility, invasion, and metastasis *in vivo*. It is interesting to note that many different signaling pathways that drive tumor growth and survival—like ERK, specific RTKs, and PKA signaling—are also important in driving mitochondrial fission through multiple independent mechanisms. This provides a very plausible explanation of why fragmented mitochondria are often found in highly invasive and metastatic cells. Lastly, many other PTMs have been identified on DRP1, MFNs, and fission receptors however, their function in the context of tumor progression remains unexplored (Table 1). Thus, an open area in this field is describing the functional consequences of these modifications on the progression to metastasis.

### Section VI: Interplay of ROS and Mitochondrial Fission in Metastasis

The role of ROS in tumor biology is well established as having both tumor promoting and tumor suppressive properties (Moloney and Cotter, 2018). The distinction between these two outcomes is determined by the level of ROS, the duration of exposure, the different species, and where it is produced spatially (Cameron et al., 2015; Moloney and Cotter, 2018; Alshaabi et al., 2021). A modest increase in ROS can drive important signaling events that promote tumor growth and metastasis however, large increases in ROS are toxic and result in cell death. In line with redox homeostasis as a recognized and crucial function of mitochondria, alteration of mitochondrial fission/fusion cycles directly influences mitochondrial and cellular ROS, which then influence many phenotypes related to metastasis.

Recently, the role of a novel mitochondrial localized fission protein—Mitochondrial 18 kDa protein (MTP18)—was described in promoting the metastatic propensity of hepatocellular carcinoma cells through mitochondrial fission and ROS production (Zhang et al., 2018). MTP18 expression is higher in hepatocellular carcinoma patients when compared to normal tissue and higher expression correlated with worse overall and recurrence-free survival. Additionally, ablation of MTP18 in hepatocellular carcinoma cell lines decreases proliferation, migration, invasion, primary tumor growth and lung metastasis *in vivo*. As expected, ablation of MTP18 showed a more fused mitochondrial network and correlated with a decrease in cellular ROS. Decreases in growth, migration, and invasion associated with depletion of MTP18 could be fully rescued through addition of exogenous H_2_O_2_. Additionally, increases in these phenotypes after MTP18 overexpression could be attenuated after treatment with a ROS scavenger, suggesting that the main mechanism of MTP18 on metastasis is through generation of ROS.

Similarly, the role of MARCH5—an E3 ubiquitin ligase known to target DRP1, MFN1, MFN2, and MiD49—was explored in the regulation of mitochondrial dynamics and ROS (Tang et al., 2019). In breast cancer, MARCH5 had higher protein expression in tumorigenic tissue and breast cancer cell lines in comparison to peritumor samples and normal immortalized breast cell lines, respectively. MARCH5 was important for promoting many phenotypes including growth, migration, invasion, primary tumor growth and lung metastasis *in vivo*. Again, MARCH5 mediated many of these phenotypes via a fragmentation of the mitochondrial pool and subsequent decreases in ROS. Changes in growth, migration, and invasion by loss or overexpression of MARCH5 could be fully rescued by treating cells with H_2_O_2_ or an ROS scavenger, respectively. While both these studies provide mostly correlative evidence between mitochondrial size and ROS production, it is evident that increases in ROS can drive metastatic phenotypes in these cells.

MiD49 dependent mitochondrial fission in ovarian cancer serves as a clear example of how changes to ROS generated by mitochondrial shape changes can drive intracellular signaling to metastatic phenotypes. As previously mentioned, in ovarian cancer cells MiD49 expression fragments the mitochondrial pool and drives tumor growth and metastasis through changes in metabolism (Zhao et al., 2020). In this context, overexpression of MiD49 increased cellular ROS and subsequently phosphorylation of AKT and mTOR (Zhao et al., 2021). Activation of AKT and mTOR could be reduced via treatment with an ROS scavenger, suggesting that fission caused by MiD49 will activate downstream signaling pathways via ROS production. Indeed, MiD49 expression drove expression and nuclear translocation of key transcription factors for fatty acid and cholesterol synthesis in an AKT dependent mechanism. This clearly demonstrates the importance ROS generated from mitochondrial fission on downstream signaling to promote metastatic phenotypes.

On the contrary, in pancreatic cancer cells ROS production from MiD49 overexpression was deleterious to growth, migration, and invasion (Bai et al., 2020). In pancreatic cancer cells, loss of growth, migratory and invasive potential associated with exogenous MiD49 expression could be fully rescued by treating cells with an ROS scavenger. This demonstrates how changes cellular ROS can result in different cellular outcomes depending on the context. As an example, in both ovarian and pancreatic cancer cells overexpression of MiD49 resulted in increased mitochondrial fission, increases in cellular ROS, and subsequent changes in tumor growth and metastasis. While in ovarian cancer cells ROS increases drove important changes in intracellular signaling to promote tumor metastasis, in pancreatic cancer cells this increase in ROS was deleterious and inhibited tumor metastasis through an unexplored mechanism.

**FIGURE 4:**
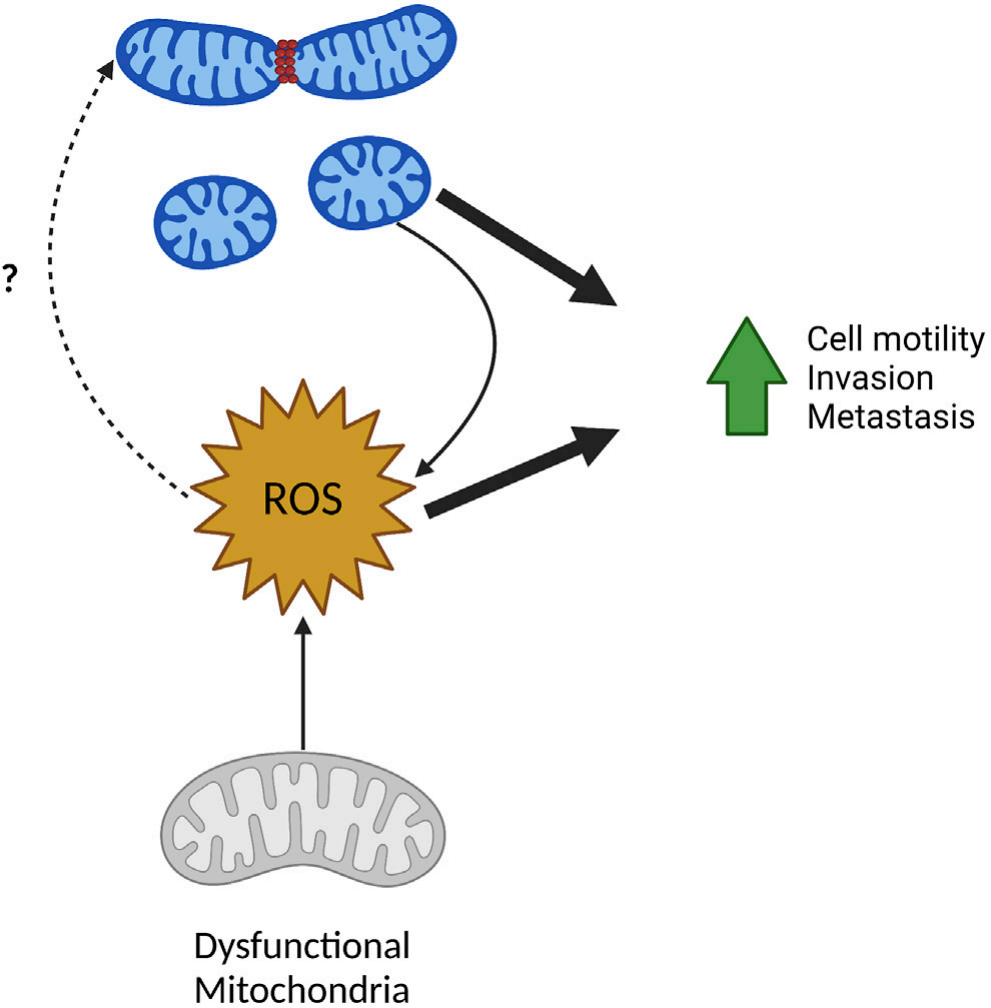
ROS in mitochondrial fission and metastasis. ROS and mitochondrial fission have a reciprocal relationship and can both drive tumor cell migration, invasion, and metastasis. Fragmented mitochondria often produce higher levels of mitochondrial and cellular ROS. Inversely, increased ROS from mitochondria with dysfunctional ETC can result in increased mitochondrial fragmentation although the mechanisms remain unclear. Created in BioRender.com.

It is clear fission of mitochondria drives ROS production and increased ROS can promote downstream changes in tumor cell-intrinsic phenotypes. Interestingly, the inverse also seems to be true where ROS can promote mitochondrial fission (Figure 4). This is demonstrated nicely by two recent publications. One group identified Isocitrate dehydrogenase 2 (NADP+) (IDH2) as a negative regulator of mitochondrial-directed tumor cell motility (Wang et al., 2019a). Using prostate cancer as a model, loss of IDH2 decreased cell growth while increasing tumor cell invasion. Consistent with *in vitro* results, cells without IDH2 showed slower primary tumor growth and increased number, but not size, of liver metastatic foci. Loss of IDH2 fragmented the mitochondrial pool consistent with an increase in pS616-DRP1 recruitment to mitochondria. Interestingly, increases in cell motility and invasion after loss of IDH2 were mediated by DRP1. Ablation of IDH2 also led to increases in mitochondrial ROS production which was critical for driving phosphorylation of S616-DRP1 and increases in cell motility. This suggests that mitochondrial dysfunction—in this case resulting from loss of IDH2—can drive DRP1 mediated increases in cell motility and invasion.

More recently a role for mitochondrial protein FUN14 Domain Containing 1 (FUNDC1), traditionally studied as a mitophagy receptor, in controlling tumor metastasis through controlling mitochondrial dynamics was explored (Li et al., 2020). Here, it was found that ablation of FUNDC1 in prostate cancer cells reduced primary tumor growth and increased metastasis to the lung and liver *in vivo*. Depletion of FUNDC1, also altered mitochondrial dynamics increasing mitochondrial trafficking to the periphery, pS616-DRP1 recruitment to mitochondria, and subsequent rates of fission. Solidifying the importance of FUNDC1 control on mitochondrial dynamics on metastatic phenotypes, increases in tumor cell invasion by loss of FUNDC1 required DRP1. Additionally, loss of FUNDC1 reduced mitochondrial ATP production and increased mitochondrial ROS in agreement with a more fragmented mitochondrial pool. Again, quenching mitochondrial ROS reduced increases in cell motility and invasion associated with loss of FUNDC1. Mechanistically, FUNDC1 interacts with AAA + protease LonP1 to promote LonP1 activity and proper folding of electron transport complex V subunits. Overexpression of LonP1 in cells without FUNDC1 restored mitochondrial ATP production, and reduced mitochondrial ROS, cell motility, and cell invasion back to basal levels. Interestingly, overexpression of LonP1 also reduced the increase pS616-DRP1 associated with loss of FUNDC1. Taken together this suggests that loss of FUNDC1 results in misfolding and subsequent decreased complex V activity, increasing mitochondrial ROS, which drives crucial changes in mitochondrial dynamics to facilitate tumor cell migration and invasion.

Both these studies show an interesting phenomenon—whereas opposed to many studies that show mitochondrial fission driving increase to ROS production—these finding show ROS production also drives mitochondrial fission. This demonstrates a reciprocal regulation mitochondrial size and ROS production, both of which positively affect many metastatic phenotypes including cell motility and invasion (Figure 4). The exact mechanism of ROS driven phosphorylation of DRP1 and subsequent mitochondrial fission is unknown and requires further investigation. As ROS is a known stimulus to activate many signaling pathways, it is possible that ROS dependent activation of kinases known to promote mitochondrial fission could serve as one explanation. As an example, MAPK pathways are known to be activated by ROS, thus activation of ERK via ROS could phosphorylate DRP1 leading to the fission of the mitochondrial pool (Son et al., 2011).

ROS is well established in its role in tumorigenesis and metastasis, however the interplay between mitochondrial fission/fusion, ROS production, and metastasis needs further explanation. Initial work demonstrates that mitochondrial fission is often associated with increases in ROS, which can impact metastatic phenotypes, however, studies linking mitochondrial fission dependent ROS production specifically affecting metastatic dissemination are lacking.

## Discussion

The reconceptualization of mitochondria as crucial signaling nodes in cancer underscores the value of therapeutically targeting these organelles. While it is clear that mitochondrial dynamics are important for tumor growth and survival, emerging literature also suggests these processes are important for metastatic dissemination of many different tumor types. Expression of fission/fusion proteins in patient samples and highly established cancer cell lines reveals that fission-promoting proteins often have higher expression in localized and disseminated tumorigenic tissue and metastatic cancer cell lines compared to normal tissue or normal immortalized cell lines, respectively (Table 2). Indeed, higher expression of these fission-promoting proteins is often associated with worse prognosis for these patients. On a cellular level, global fragmentation of mitochondria in cancer cells drives a multitude of phenotypes including changes in metabolism, increased ROS, increased trafficking of mitochondria to the cell periphery, and increased membrane protrusions that promote migration, invasion, and metastasis of these cells (Figure 2). Finally, in addition to changes in expression we have discussed several signaling pathways that converge to promote mitochondrial fission and drive metastatic dissemination (Figure 3). Thus, commonly dysregulated signaling pathways observed in cancer also promote mitochondrial fission providing an explanation of why metastatic cancer cells often have highly fragmented mitochondrial pools.

Despite these new advances, there are many open questions that remain in this field. While many large-scale studies observe increased expression of fission-promoting proteins and associated with worse prognosis, we still have a poor understanding of how expression of these genes are dysregulated in metastatic cancer. Furthermore, while most literature in the context of metastasis shows smaller mitochondria associated with a more metastatic phenotype—suggesting that there is either an increase in mitochondrial fission or a decrease in mitochondrial fusion—it is important to remember that mitochondrial shape changes are a continuous process. Indeed this concept is emphasized as several reports argue that there may be an increase in both fission and fusion processes even amongst a fragmented mitochondrial pool (Caino et al., 2016; Wang et al., 2019a). It is interesting to speculate that the ability for mitochondria to be able to rapidly change shapes in order to react to different environmental stimuli may be highly advantageous to the cell. With the emergence of many novel microscopy techniques for detecting real time mitochondrial shape changes, it will be important going forwards to use these novel techniques to determine changes in mitochondrial fission/fusion cycles rather than a simple snapshot of the total pool of mitochondria in a cell at a given time (Bertolini et al., 2021; Lefebvre et al., 2021).

Amongst PTMs on mitochondrial fission and fusion proteins, pS616-DRP1 remains the most well characterized in tumor progression. However, in terms of other PTM regulation of the fission/fusion GTPases and their receptors in tumor progression virtually nothing is known (Table 1). Strengthening the importance of investigating novel PTMs, recent research identifying a new and critical PTM on FIS1 by MET demonstrates the necessity of this signaling pathway to drive metastatic dissemination in hepatocellular carcinoma (Yu et al., 2021). Finally, the relevance of mutations identified from patient samples could yield new and interesting insight into the regulation of fission and fusion.

Another emerging area is the importance of mitochondrial fission/fusion in stromal or tumor microenvironmental cell types that promote metastasis. It is well known that the tumor microenvironment plays a critical role for tumor growth and metastasis. While mitochondrial shape changes have been observed in many other cell types very little is known about the impact of this process on metastasis. As an example, the importance in mitochondrial fission/fusion has been demonstrated in NK cells and T-cells, however the functional importance of this in the context of metastasis remains unknown (Buck et al., 2016; Zheng et al., 2019). Furthermore, a recent study demonstrates how secretion of extracellular vesicles from tumor cells can influence mitochondrial dynamics and tumorigenesis in surrounding normal cells (Bertolini et al., 2020). Finally, recent research demonstrates targeting OPA1 in the endothelial cell compartment can inhibit metastatic dissemination underscoring the importance of studying mitochondrial dynamics in other cell types (Herkenne et al., 2020). Exploration into these exciting new areas will hopefully serve as the basis to generate novel therapeutics to target metastatic disease.
